# Functional and Aesthetic Restoration of Amputated Fingers Using Osseointegrated Dental Implants: Case Presentations

**DOI:** 10.7759/cureus.105273

**Published:** 2026-03-15

**Authors:** Ahmad Almigdad, Ahmad Alzoubi, Iasmina Alhiasat

**Affiliations:** 1 Department of Orthopedics, Jordanian Royal Medical Services, Amman, JOR; 2 Department of Dentistry, Jordanian Royal Medical Services, Amman, JOR

**Keywords:** dental implants, digital prosthesis, finger amputation, osseointegration, silicon prosthesis

## Abstract

Traumatic finger amputations significantly impair hand function and aesthetic appearance. Conventional prosthetic options are often limited when the residual stump is inadequate for secure prosthesis retention. Osseointegrated dental implants offer a promising alternative for anchoring custom-made prostheses. This case series presents three patients: Patient 1 was a 23-year-old male with traumatic disarticulation at the proximal interphalangeal joint of the right ring finger; Patient 2 was a 40-year-old male with traumatic amputation of the right index finger at the level of the middle phalanx; and Patient 3 was a 26-year-old female with traumatic amputation of the right middle finger at the level of the head of the middle phalanx.

Each patient underwent preoperative radiographic evaluation to assess the quantity and quality of the residual bone and to determine the appropriate implant length and diameter. A two-stage surgical approach was employed, consisting of initial implant placement followed by attachment of healing abutments and subsequent prosthetic fitting. All patients achieved satisfactory osseointegration and favorable functional outcomes, with good prosthetic stability and cosmetic results. One patient developed a superficial infection around the abutment, which resolved with oral antibiotic therapy. Osseointegrated implants represent a viable alternative for digital amputees when reconstructive procedures are contraindicated, providing reliable retention, enhanced functionality, and a natural appearance.

## Introduction

Finger amputation carries a significant functional and psychological impact on patients [1]. Surgical reconstruction techniques, including toe-to-hand transfer, local or free flap reconstruction, and procedures such as pollicization (the surgical creation of a functional thumb from another digit), can restore partial function but are technically demanding and not suitable for all patients. These procedures require specialized microsurgical expertise, prolonged rehabilitation, and may result in donor-site morbidity or unsatisfactory cosmetic outcomes. For patients who are not candidates for complex reconstruction, prosthetic rehabilitation remains an important alternative [2,3]. Conventional silicone finger prostheses are typically retained by soft-tissue suspension over the residual stump. While they can provide acceptable cosmetic restoration, their functional utility is often limited. These prostheses may become loose during daily activities, lack stability during grasping, and can cause skin irritation, perspiration accumulation, or discomfort. Furthermore, retention becomes particularly challenging in patients with short or irregular residual stumps [4]. Bone-anchored prostheses, using dental implants placed at the amputation level, offer several advantages, including improved function, enhanced cosmetic outcomes, and better sensory-proprioceptive perception [5].

Osseointegration, a biological process in which a titanium implant forms a direct structural and functional connection with living bone, was originally developed in dental implantology for anchoring dental prostheses. This principle has since been adapted for limb prosthetic applications. In the context of digital reconstruction, a dental implant can be inserted into the remaining phalanx, allowing attachment of an external abutment that securely anchors a custom-made silicone prosthesis. This bone-anchored system improves prosthetic stability and may provide enhanced proprioceptive feedback, a phenomenon sometimes referred to as osseoperception. The procedure is performed in two stages: first, a dental implant is inserted into the bone, followed by a three-month period to allow complete osseointegration. In the second stage, an anchoring abutment is attached, enabling retention of a custom-made silicone prosthesis that matches the patient’s finger in shape, size, and color [6].

Although osseointegrated implants have been increasingly reported as a promising option for digital prosthetic retention, the available literature remains relatively limited. In this study, we present three cases of traumatic finger amputations at different levels managed using osseointegrated dental implants for prosthetic retention. Our aim is to demonstrate the clinical feasibility of this approach and to highlight its functional and aesthetic outcomes in patients who were not candidates for conventional reconstructive procedures.

## Case presentation

This prospective case series was conducted at the Royal Rehabilitation Center in Amman, Jordan, from September 2024 to January 2026 and included all consecutive patients who met the eligibility criteria for osseointegrated dental implant reconstruction. Three patients with traumatic finger amputations at different levels underwent reconstructive surgery using osseointegrated dental implants. Each patient underwent preoperative radiographic evaluation to assess the quantity and quality of the residual bone and to determine the appropriate implant length and diameter. A two-stage surgical approach was employed, consisting of initial implant placement followed by attachment of healing abutments and subsequent prosthetic fitting. The following cases describe the surgical procedure, postoperative course, and functional outcomes for each patient.

Case 1

**Figure 1:**
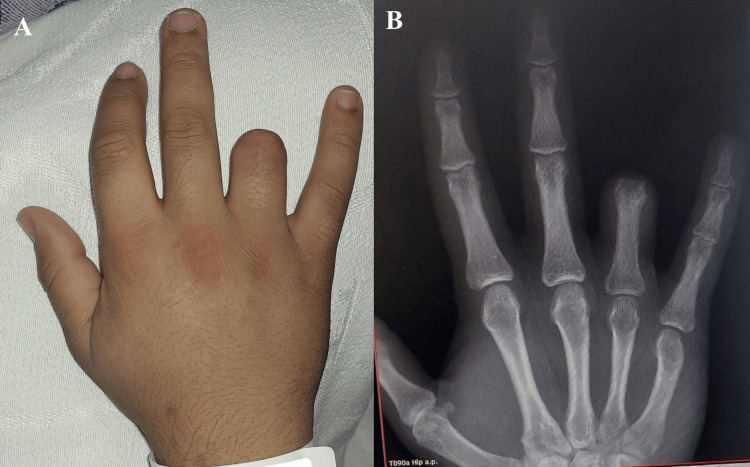
Clinical (A) and radiographic (B) images demonstrating disarticulation at the level of the PIPJ. PIPJ: Proximal interphalangeal joint.

A 23-year-old male presented with a traumatic disarticulation at the proximal interphalangeal (PIP) joint of the right ring finger, sustained three years earlier (Figure 1). There were no associated injuries. The patient reported dissatisfaction with the cosmetic appearance of the amputated finger and mild functional limitations during daily activities, particularly when grasping small objects. He had not previously used a prosthetic device but expressed interest in a stable prosthetic solution that could restore finger length and improve the appearance of the hand. Preoperative measurements of the inner cortical width and the available bone length from the amputation site to the subchondral bone were obtained. These values guided selection of the appropriate implant size.

**Figure 2:**
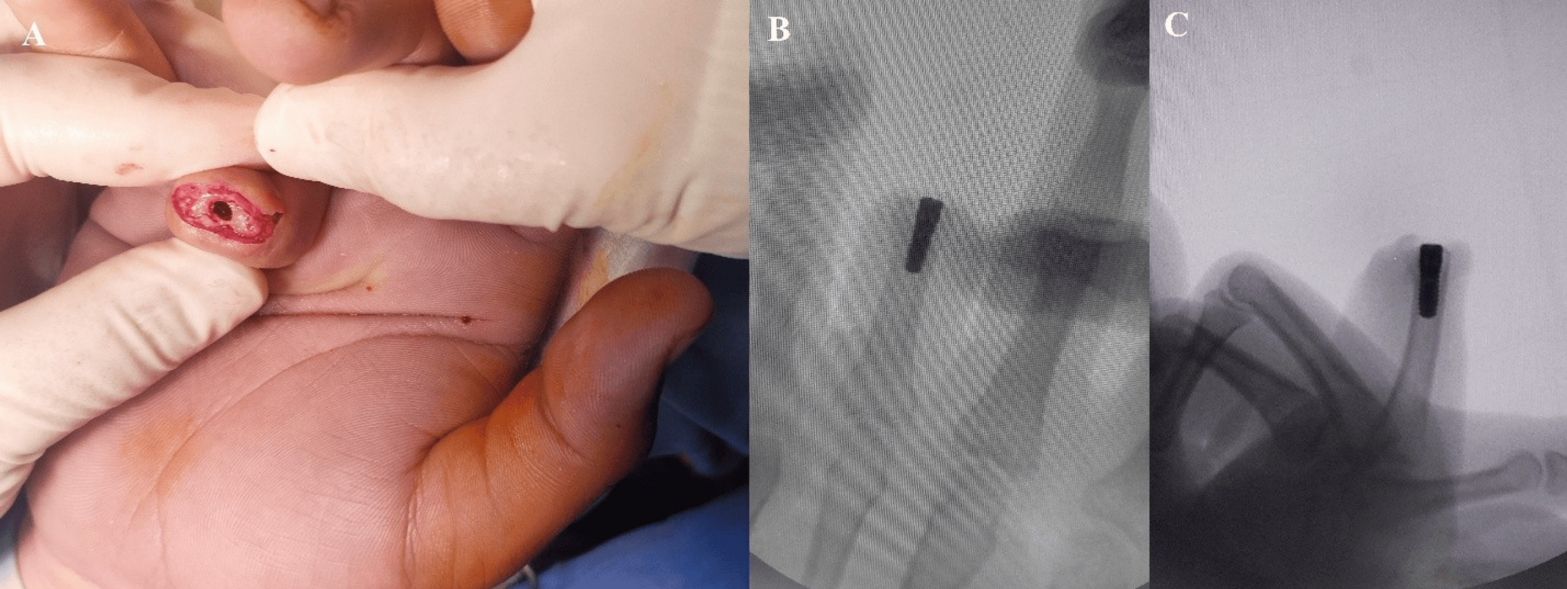
(A) Intraoperative image showing preparation of the medullary canal of the proximal phalanx for dental implant insertion. (B, C) Radiographic images demonstrating appropriate positioning of the dental implant.

In the first stage of surgery, a titanium dental implant measuring 14 mm in length and 4.5 mm in diameter was inserted into the proximal phalanx of the ring finger (Figure 2). The patient was monitored with serial radiographs to assess osseointegration.

**Figure 3:**
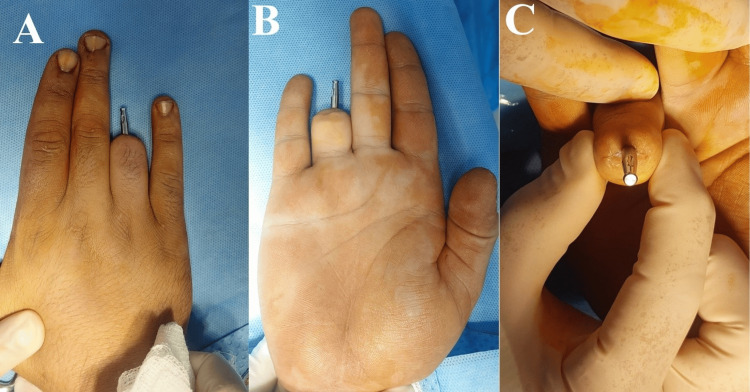
(A-C) Custom-made abutment fixed to the dental implant, with good healing of the surrounding soft tissue.

**Figure 4:**
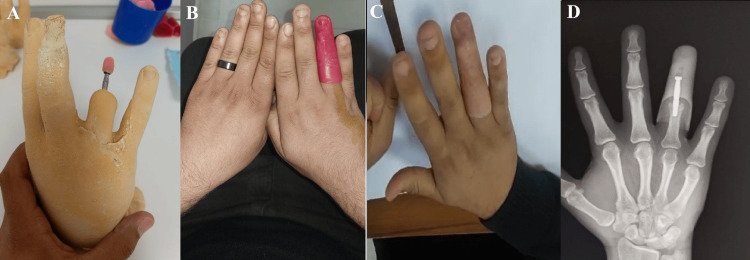
(A) Hand cast model with the abutment in position for fabrication of a retained prosthesis. (B) Initial retained prosthesis. (C) Final silicone prosthesis in situ. (D) Radiographic image demonstrating the connection between the abutment and the dental implant, with the prosthesis in place.

Three months later, the second stage of surgery was performed. The skin over the implant was re-elevated, and healing caps were attached (Figure 3). After a two-week soft-tissue healing phase, impression cylinders were fitted, and final impressions were obtained using silicone impression material (Figure 4). A custom silicone prosthetic finger was fabricated and attached to the implant abutment.

Following prosthetic fitting, the patient was followed for 11 months, during which he reported improved confidence in the appearance of the hand and was able to perform daily activities with greater ease because of the stability of the prosthesis.

Case 2

**Figure 5:**
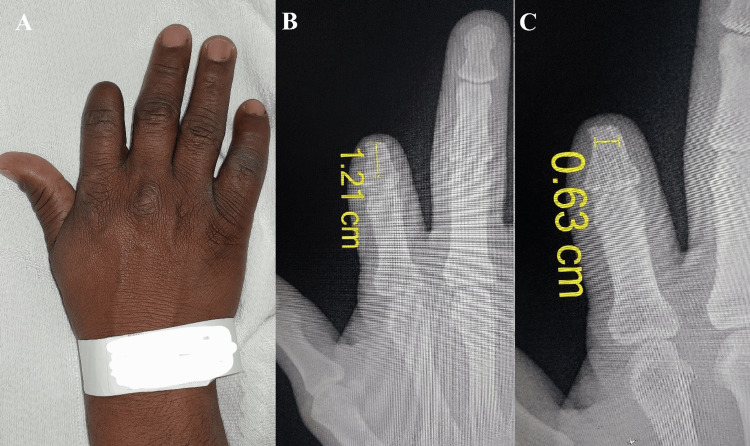
(A) Clinical image showing amputation at the level of the middle phalanx of the index finger. (B, C) Radiographic images demonstrating amputation at the level of the middle phalanx of the index finger.

A 40-year-old male presented with a traumatic amputation of the right index finger caused by a mechanical meat grinder. The injury resulted in loss of the digit at the mid-shaft of the middle phalanx (Figure 5). The patient presented four years after the incident. Preoperative radiographs of the stump revealed a bone width of 6.3 mm and a length of 12.1 mm, sufficient for implant placement. Based on these findings, a suitable dental implant was selected.

The patient reported difficulty performing certain manual tasks due to the shortened digit and expressed concern regarding the cosmetic appearance of the hand. He had not previously used a conventional silicone prosthesis because of concerns regarding poor retention and discomfort. At presentation, the patient demonstrated preserved motion of the remaining finger joints and maintained overall hand function.

**Figure 6:**
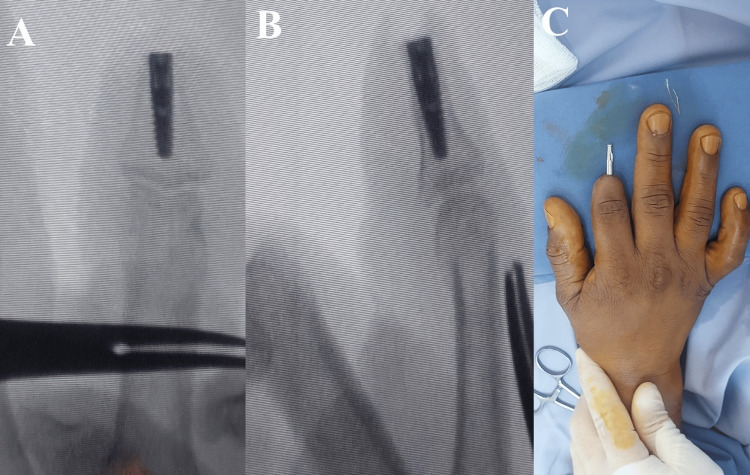
(A, B) Radiographic images demonstrating proper positioning of the dental implant within the middle phalanx. (C) Intraoperative image from the second-stage surgery showing fixation of the abutment to the dental implant.

During the first surgical stage, a dental implant measuring 12 mm in length and 3.5 mm in diameter was placed into the middle phalanx of the index finger (Figure 6). Radiographic follow-up confirmed appropriate positioning and successful osseointegration.

**Figure 7:**
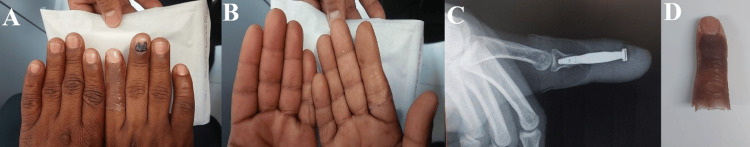
(A) Dorsal and (B) palmar views of both hands showing the final retained silicone prosthesis of the right index finger in situ. (C) Radiographic image demonstrating the connection between the abutment and the dental implant, with the prosthesis in place. (D) Silicone prosthesis.

At four months postoperatively, the second stage of surgery was performed. The implant site was reopened, healing caps were placed, and two weeks later, impression cylinders were secured. Final impressions were made using silicone material to fabricate a custom digital prosthesis, which was successfully fitted (Figure 7). The patient reported improvement in both hand appearance and functional performance during daily activities such as grasping objects.

During postoperative follow-up, the patient was monitored for one year. Early in the course, he developed a superficial infection around the abutment site, presenting with localized erythema and mild discharge, which was successfully managed with a short course of oral antibiotics and local wound care. At the one-year follow-up, the implant remained stable, the prosthesis demonstrated good functional performance, and the patient reported high satisfaction with both function and appearance.

Case 3

**Figure 8:**
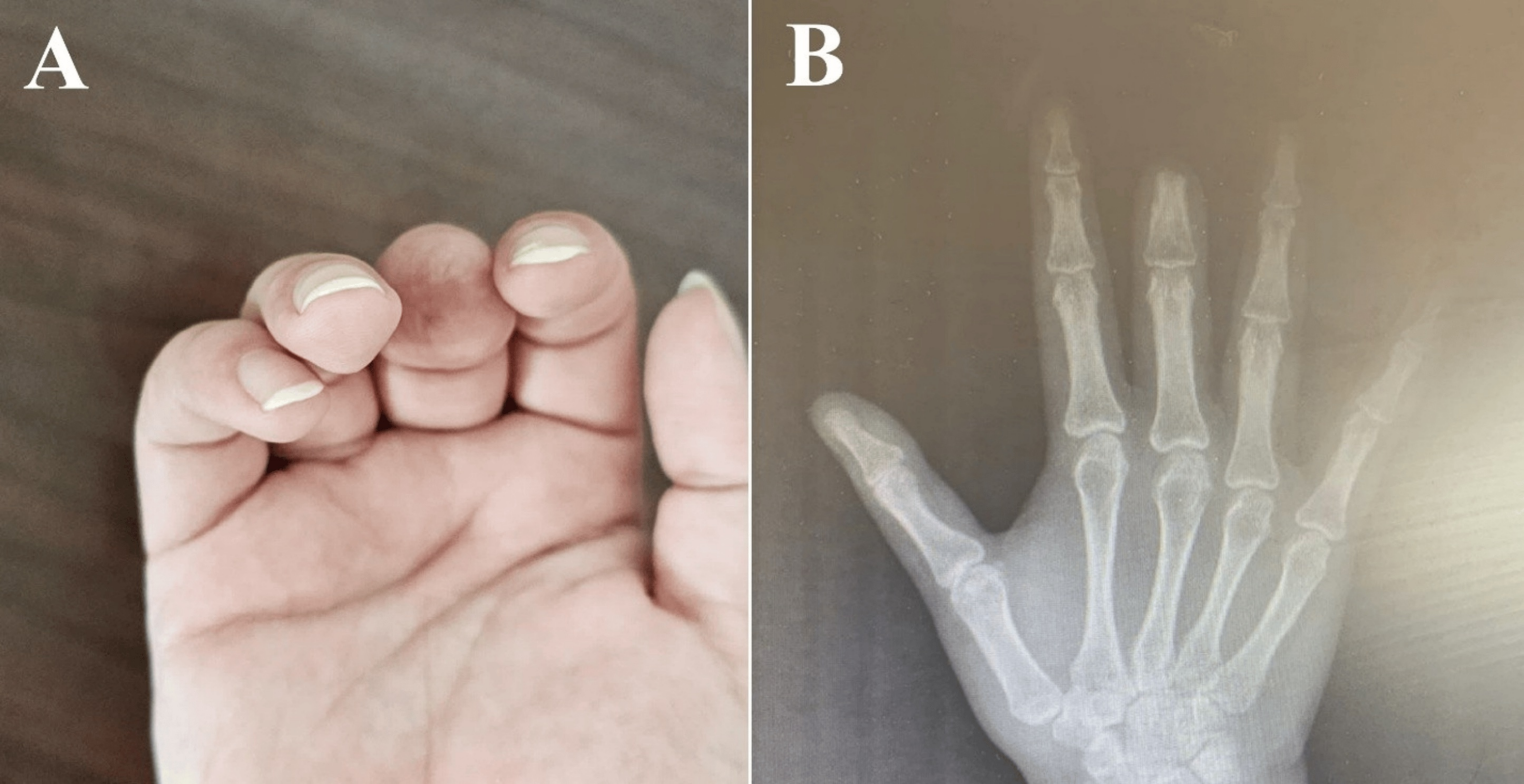
(A) Clinical image showing amputation at the level of the middle phalanx of the middle finger. (B) Radiographic image demonstrating amputation at the level of the head of the middle phalanx of the middle finger.

A 26-year-old female presented with a history of traumatic amputation of the right middle finger at the level of the head of the middle phalanx, sustained in a road traffic accident 2.5 years earlier (Figure 8). The patient sought treatment primarily because of dissatisfaction with the aesthetic appearance of the hand and the psychological impact of the missing finger during social interactions. She expressed a strong desire for a prosthetic solution that could restore the natural appearance of the digit. At presentation, she demonstrated full range of motion and preserved function in the affected hand.

**Figure 9:**
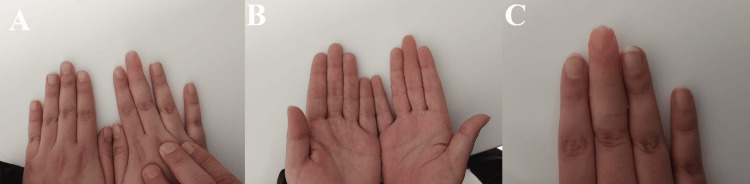
(A-C) Clinical images demonstrating the retained silicone prosthesis in situ.

In the first surgical stage, a dental implant measuring 14 mm in length and 4 mm in diameter was placed into the residual middle phalanx of the middle finger. Osseointegration was monitored over time. Three months later, the second surgical stage was completed with exposure of the implant and attachment of healing caps. The final prosthesis fabrication process was initiated shortly afterward (Figure 9). Following prosthetic fitting, the patient was followed for 9 months. During this period, the prosthesis demonstrated good retention and stability during routine hand activities, resulting in good functional recovery and a satisfactory cosmetic outcome. The patient reported high satisfaction with both the appearance and function of the prosthesis.

Table 1 presents key clinical and procedural data for all three cases, including implant details, complications, and functional outcomes, providing a clear and structured comparison to enhance understanding and reproducibility of the procedure.

**Table 1:** Summary of patient characteristics, implant details, and outcomes.

Case	Age / Sex	Level of Amputation	Implant Dimensions (mm)	Follow-up Duration (months)	Complications	Functional Outcomes / Patient Satisfaction
1	23 / M	Right ring finger disarticulation at the proximal interphalangeal (PIP) joint	14 × 4.5	11	None	Good stability and satisfactory cosmetic outcome; patient reported being able to perform daily activities
2	40 / M	Right index finger amputation at the mid-shaft of the middle phalanx	12 × 3.5	12	Superficial infection	Stable prosthesis and improved function; patient reported satisfaction
3	26 / F	Right middle finger amputation at the level of the head of the middle phalanx	14 × 4	9	None	Good functional recovery and satisfactory cosmetic outcome; patient reported high satisfaction

## Discussion

In cases of finger amputation where replantation is not possible and reconstructive surgery is not feasible or is declined, prosthetic rehabilitation becomes a practical and valuable alternative. Conventional silicone finger prostheses offer some cosmetic and functional benefits; however, they are often limited by poor retention, lack of tactile feedback, perspiration accumulation, and the need for frequent replacement because of skin irritation or mechanical wear. In contrast, osseointegrated implants provide several advantages, including stable, bone-anchored retention that improves both prosthetic control and cosmetic appearance. Additionally, these implants can facilitate partial sensory feedback, known as osseoperception, which enhances grip stability and proprioceptive awareness [6].

Our case series contributes to the growing body of evidence supporting the use of dental implants for digital prosthetic retention. Consistent with the findings of Aydin C et al. [7] and Sierakowski A et al. [8], our patients achieved favorable cosmetic outcomes and functional restoration with minimal complications. Notably, the enhanced stability of the prostheses allowed patients to perform daily activities with greater ease and renewed confidence.

Regarding the retained prosthesis, it was fabricated in a straight alignment, which restricted the finger’s ability to contribute effectively to grip, especially during flexion. Thus, we suggest refabricating the prosthesis in a more flexed position to improve functional grasp. The color mismatch was acceptable to the male patients but not to the female patient. Importantly, the prosthesis color remained stable under varying weather and temperature conditions, whereas the surrounding skin changed with temperature fluctuations.

Furthermore, our outcomes align with those reported by Li AT et al. [9], who found that although osseointegrated prostheses may offer slightly reduced sensory feedback and range of motion compared with replantation, overall patient satisfaction and functional outcomes were comparable. Importantly, osseointegration presents a dependable long-term solution with fewer complications for patients who are not candidates for complex microsurgical reconstruction.

Proper patient selection and surgical technique are critical for achieving optimal outcomes. Patients with adequate bone stock and realistic expectations tend to experience the most favorable results. However, complications such as abutment loosening, cold intolerance, and soft-tissue irritation can occur [8]. In most cases, these issues are manageable and do not significantly affect the use or functionality of the prosthesis.

The procedure can be performed using either a one-stage or two-stage approach, both of which have been reported in the literature to yield favorable outcomes [10]. The one-stage approach offers the advantage of reduced overall treatment time and fewer hospital visits. However, the two-stage technique remains the preferred option in cases with compromised bone quality or complex soft-tissue conditions, as was the protocol followed in our study.

Although our early results are encouraging, osseointegration is not without its challenges. The procedure requires at least two surgical stages, involves a risk of infection, and demands a prolonged rehabilitation period. Despite these limitations, the functional improvements and psychological benefits observed in our patients highlight the significant value of osseointegration as a reconstructive option for appropriately selected individuals with digital amputations.

Using a standard dental implant increases the risk of loosening and periprosthetic fracture, as the long retained prosthesis places significant mechanical load on the short implant stem. We recommend using a custom-made, longer implant to reduce this risk.

## Conclusions

In appropriately selected patients with adequate residual bone stock and chronic single-finger amputations, osseointegrated dental implants represent a technically feasible option for prosthetic finger reconstruction. Clinical observation in these three cases suggests that the implants may provide stable prosthetic retention and may improve both function and appearance, although formal outcome measurements were not performed. Soft-tissue complications, such as periprosthetic infection, can occur and require monitoring and prompt management. These preliminary findings support further investigation in larger prospective studies with standardized functional and patient-reported outcome measures to better evaluate efficacy, safety, and long-term durability.
